# The Precision Frontier: Revolutionising Head and Neck Cancer Management Through Theranostics, Liquid Biopsy, and AI-Powered Imaging

**DOI:** 10.3390/cancers17233792

**Published:** 2025-11-27

**Authors:** Muy-Teck Teh

**Affiliations:** Centre for Oral Immunobiology & Regenerative Medicine, Institute of Dentistry, Barts & The London School of Medicine and Dentistry, Queen Mary University of London, London E1 2AT, UK; m.t.teh@qmul.ac.uk

**Keywords:** head and neck cancer, theranostics, liquid biopsy, AI-powered imaging, early diagnosis, oral cancer, oral squamous cell carcinoma, proliferative verrucous leukoplakia, peri-implant malignancy

## Abstract

Head and neck cancer (HNC) diagnostics are undergoing a transformative shift. Recent research published in *Cancers* highlights a paradigm shift in the comprehensive management of HNC, driven by precision oncology and disruptive technologies. AI-enhanced imaging and non-invasive biomolecular fingerprinting are redefining early detection, with tools like infrared spectroscopy and hyperspectral imaging delivering near-perfect accuracy and real-time surgical guidance. Liquid biopsy is emerging as a powerful surveillance modality, capable of detecting recurrence months before conventional imaging and offering prognostic insights via cell-free DNA analysis. Theranostic agents in nuclear medicine show promise for rare HNC subtypes, though broader molecular targets remain a challenge. These technologies may have utility for complex presentations such as proliferative verrucous leukoplakia (PVL)-associated oral squamous cell carcinoma (OSCC), which disproportionately affects women, and peri-implant OSCC, which is often misdiagnosed and requires aggressive intervention. Collectively, these innovations directly address long-standing challenges: early detection, accurate staging, treatment personalization, monitoring of minimal residual disease and timely cancer care—where diagnostics not only inform treatment but actively shape outcomes. This editorial underscores the urgency of integrating such tools into clinical pathways to improve survival and quality of life for HNC patients globally.

## 1. Introduction

Head and neck cancer (HNC), a diverse group of malignancies affecting the upper aerodigestive tract, remains a formidable global health challenge, ranking the third most prevalent cancer worldwide [1]. While current diagnostic standards, primarily tissue biopsy and traditional imaging, offer critical information, they often fall short by being invasive, providing only a static assessment of the disease, and suffering from subjectivity or poor speed [2].

However, recent research published in *Cancers* demonstrated an unprecedented convergence of nuclear medicine, molecular diagnostics, and artificial intelligence (AI), promising a definitive shift toward personalised and precision oncology in HNC management. Innovations in theranostics [3], sophisticated liquid biopsy techniques [4,5], and real-time AI-powered surgical imaging [6,7] are reshaping the care continuum, offering hope for earlier diagnosis, more precise treatment, and improved patient outcomes.

## 2. Expanding Therapeutic Horizons with Theranostics

Theranostics is an emerging approach in cancer care that uses one radioactive compound to determine the effectiveness of a chemically similar agent for both imaging and treatment. While the idea originated decades ago (e.g., with Iodine-131 in 1936), recent advancements, such as the FDA approval of ^177^Lu-DOTATATE (Lutathera) in 2018, have brought the field to the forefront [8]. Oldan et al. [3] reviewed and highlighted current and emerging theranostic drug pairs for head and neck tumours, summarizing clinical studies and exploring future candidates not yet tested in humans.

In HNC specifically, research is focused on extending the use of established agents and developing new ones, despite the challenge that the most common tumour subtype, squamous cell carcinoma (SCC), currently lacks a specific targeted agent. The best results so far have been seen with Somatostatin Receptor (SSTR) Inhibitors, such as DOTATATE. These agents target SSTRs expressed on neuroendocrine tumours. Early studies involving dozens of patients with head and neck paragangliomas and progressive meningiomas demonstrate promising results, showing a mixture of partial response and stabilization of disease, similar to outcomes observed in midgut neuroendocrine tumours. Research is ongoing, with suggestions that intraarterial administration of DOTATATE might enhance efficacy in meningioma treatment.

The picture is less clear for Prostate-Specific Membrane Antigen (PSMA) agents and Fibroblast-Activating Protein (FAP) inhibitors. PSMA-targeted agents, highly successful in prostate cancer, are being explored for salivary tumours (like adenoid cystic carcinoma) and iodine-refractory thyroid cancer. While case reports hinted at initial success, early trials generally show disease stabilization at best for salivary gland cancer. FAP agents, which target the tumour microenvironment (cancer-associated fibroblasts), have shown a mix of partial response and stabilization in differentiated thyroid cancer (DTC) and are under continued study.

## 3. AI and Spectroscopy: New Tools for Objective Diagnosis

Conventional histopathology, the current standard for confirmatory diagnosis, is challenged by high subjectivity and variability, particularly when assessing precursor lesions like oral epithelial dysplasia (OED). This limitation is driving the integration of AI with advanced spectral imaging technologies to provide objective, quantitative analysis as reviewed by Alajaji et al. [6].

Infrared (IR) spectroscopy, a label-free technique that generates biochemical profiles or “biomolecular fingerprints” of tissues, is proving highly sensitive for detecting subtle molecular alterations. When coupled with Machine Learning (ML) techniques—such as Linear Discriminant Analysis (LDA) and Support Vector Machines (SVM)—it shows exceptional diagnostic capability for oral cancer. Studies have reported highest sensitivity and specificity of 100%, with accuracy reaching 95–96%, for oral cancer diagnosis applications. Even for prognostic applications concerning malignant transformation of OED, sensitivity and specificity were reported up to 84% and 79%, respectively [6].

A parallel advancement is occurring in the operating room with Hyperspectral Imaging (HSI) combined with Deep Learning (DL) for real-time intraoperative decision-making, as elegantly demonstrated by Bali et al. [7]. HSI captures comprehensive spectral information across multiple wavelengths, enabling the discrimination of biological tissues based on their unique spectral signatures. Bali et al. [7] demonstrated a novel workflow integrating multi-angle HSI data with 3D histological models (serving as histopathological ground truth) to train Convolutional Neural Networks (CNNs) for tumour margin assessment in HNSCC. This system achieved an impressive overall classification accuracy of 0.98 and a tumour sensitivity of 0.93 for detecting tumour regions. Crucially, this label-free approach allows for rapid analysis, providing surgeons with feedback in approximately 10 min, contrasting favourably with the standard 30 min processing time and lower accuracy of frozen section histology. The integration of HSI data into a spatially coherent 3D model, potentially using Augmented Reality (AR), further enhances surgical interpretability and precise anatomical orientation.

## 4. Liquid Biopsy: Real-Time Molecular Surveillance

Liquid biopsy for the analysis of disease biomarkers, from either non-invasive saliva, urine and other secreted bodily fluids or minimally invasive blood samples, represents a paradigm shift for disease monitoring. This technique has been demonstrated to be impactful in HNC, with utility for human papilloma virus (HPV)-associated HNC recently reviewed in Parisi et al. [5].

For HPV-positive HNSCC, which is biologically and clinically distinct, viral DNA sequences offer highly specific markers for detection and monitoring. Liquid biopsy, specifically focusing on circulating cell-free HPV DNA, has demonstrated high sensitivity (90–95%) and specificity (>98%) in detection. Longitudinal monitoring of circulating HPV DNA levels is a robust predictor of treatment response and disease recurrence. Studies show that liquid biopsy can detect recurrence on average 3 to 6 months earlier than conventional imaging. Between available methods, droplet digital PCR (ddPCR) is often recommended for routine clinical monitoring due to its high sensitivity (>95%) and specificity (>98%), coupled with superior cost-effectiveness compared to next-generation sequencing (NGS).

In largely HPV-negative OSCC, cfDNA analysis is also proving valuable for diagnosis and prognosis as demonstrated by Bhattacharya et al. [4], revealing significant genomic alterations. Elevated cfDNA concentration in OSCC patients is observed to be significantly higher than in healthy individuals, and critically, a high concentration is significantly associated with an advanced tumour stage (stage 3 or 4), increased recurrence risk, and reduced survival probability. Deep genome sequencing of cfDNA has uncovered significant findings, including copy number variations (CNVs) and single-nucleotide variants (SNVs) like MUC3A, MUC6, and KMT2C. Notably, Bhattacharya et al. [4] identified and validated a novel fusion gene, TRMO-TRNT1, associated with late-stage tumours, and sequences of various oncogenic viruses (including HPV, HSV, simian virus, and enterovirus) integrated into the cfDNA. These findings establish cfDNA as a powerful, non-invasive biomarker for diagnostics and personalized therapeutic strategies in OSCC.

## 5. Addressing High-Risk Subtypes and Diagnostic Challenges

Two recent research articles highlighted the critical need to distinguish between OSCC subtypes based on aetiology and clinical presentation:

1. Proliferative Verrucous Leukoplakia (PVL)-associated OSCC [9]: PVL is an oral potentially malignant disorder (OPMD) with the highest rate of malignant transformation (43.87–65.8%). OSCC preceded by PVL (OSCC-PVL) exhibits distinct clinical characteristics compared to conventional OSCC (OSCC-noPVL). OSCC-PVL is much more frequent in women (70% vs. 34.4% in OSCC-noPVL) and less often associated with tobacco use. It commonly presents in less aggressive clinical forms (e.g., erythroleukoplakia type 30% vs. 63.3% of the more clinically aggressive ulcerated form predominated in OSCC-noPVL) and, critically, is associated with significantly more frequent early T stages (T1 and T2 in 80% of cases) than OSCC-noPVL (57.78% of cases) [9].

2. Peri-Implant OSCC [10]: Another complex challenge is the diagnosis of Peri-Implant OSCC. This malignancy is frequently misdiagnosed as peri-implantitis due to its erythroplakia-like or leuko-erythroplakia-like clinical and radiological presentation. Peri-implant OSCC occurred most often in patients who already had risk factors, specifically Oral Potentially Malignant Disorders (OPMDs) (52.4% of patients) or a prior history of OSCC (14.3%). Diagnosis is further complicated by the scattering caused by dental implants, which impairs CT imaging of the bone-implant interface, necessitating the use of alternative imaging modalities like MRI. The aggressive nature of this malignancy often requires demolitive surgery rather than conservative excision, and patient prognosis strictly depends on the grade of the cancer (with poorly differentiated tumours having a worse outlook) [10].

## 6. Conclusions

The convergence of theranostics, AI-driven spectroscopy, and liquid biopsy is paving the way for a new era of precision in HNC management. Liquid biopsy offers unparalleled potential for early detection and prognostic stratification in HNSCC (including OSCC), coupled with highly sensitive recurrence monitoring for HPV-positive disease. Simultaneously, advanced optical imaging (HSI) promises to deliver real-time, objective tumour delineation, enhancing surgical precision and efficiency. To fully realize these promises, the field must overcome persistent challenges, most notably the requirement for larger, diverse cohorts to validate ML and DL models and ensure the robustness and generalizability of findings. Continued research focused on standardizing analytical protocols, optimizing cost-effective hybrid approaches (like combining ddPCR and NGS), and integrating these advanced diagnostics into routine clinical workflow will be essential for transforming patient outcomes in HNC. As innovation accelerates in HNC diagnostics and therapeutics, the field must remain grounded in cost-effectiveness, scalability, and equitable access—especially for underserved populations in resource-limited settings where the need is greatest.
